# Glycogen

**Published:** 1872-12

**Authors:** 


Glycogen.—M. Claude Bernard announces that he has recommenced his studies on the evolution of glycogen in the eggs of birds. He recalls to mind that in 1848 he discovered sugar (*glycose*) in the liver of animals; that in 1855 and 1857 he proved that this sugar is derived from a particular source—glycogen—fixed in the hepatic tissue. Finally, in 1859, he again found this glycogen in the placental organs of mammals and in the vitelline membrane of birds. It is at this point that he recommences the research.—*Ex.*

